# Propionate Induces Energy Expenditure via Browning in Mesenteric Adipose Tissue

**DOI:** 10.1210/clinem/dgaf280

**Published:** 2025-05-12

**Authors:** Baichen Lu, Aylin C Hanyaloglu, Yue Ma, Adam E Frampton, Christopher Limb, Nabeel Merali, Madhava Pai, Rehan Ahmed, Mark Christian, Gary Frost

**Affiliations:** Section of Nutrition, Department of Metabolism, Digestion and Reproduction, Faculty of Medicine, Imperial College London, London W12 0NN, UK; Institute of Reproductive and Development Biology (IRDB), Department of Metabolism, Digestion, and Reproduction, Faculty of Medicine, Imperial College London, London W12 0NN, UK; Section of Nutrition, Department of Metabolism, Digestion and Reproduction, Faculty of Medicine, Imperial College London, London W12 0NN, UK; Section of Oncology, Department of Clinical and Experimental Medicine, Faculty of Health and Medical Sciences, The Leggett Building, University of Surrey, Guildford, Surrey GU2 7WG, UK; Department of Surgery and Cancer, Faculty of Medicine, Imperial College London, London W12 0NN, UK; Section of Oncology, Department of Clinical and Experimental Medicine, Faculty of Health and Medical Sciences, The Leggett Building, University of Surrey, Guildford, Surrey GU2 7WG, UK; Section of Oncology, Department of Clinical and Experimental Medicine, Faculty of Health and Medical Sciences, The Leggett Building, University of Surrey, Guildford, Surrey GU2 7WG, UK; Department of Surgery and Cancer, Faculty of Medicine, Imperial College London, London W12 0NN, UK; Department of Surgery and Cancer, Faculty of Medicine, Imperial College London, London W12 0NN, UK; Department of Biosciences, School of Science and Technology, Nottingham Trent University, Clifton, Nottingham NG11 8NS, UK; Section of Nutrition, Department of Metabolism, Digestion and Reproduction, Faculty of Medicine, Imperial College London, London W12 0NN, UK

**Keywords:** adipose tissue, SCFA, propionate, browning, thermogenesis

## Abstract

**Context:**

Short-chain fatty acids, such as propionate, are produced from the fermentation of dietary fiber by gut microbiota and modulate adipose tissue (AT) metabolism to influence whole-body metabolic processes. Abdominal AT, critical in glucose and lipid homeostasis, is categorized into mesenteric, omental, and subcutaneous types based on its location. ATs display different metabolic phenotypes due to their distinct adipocyte lineages—white, brown, and beige. Recent evidence points to a significant effect of propionate on abdominal AT.

**Objective:**

Our study investigated the actions of propionate on the 3 types of human abdominal AT.

**Methods:**

AT from distinct depots (mesenteric, omental, and subcutaneous) were collected from 40 patients who underwent open abdominal surgery for cholecystectomy or explorative laparotomy. Tissue explants and isolated adipocytes were treated with 1 mM propionate to assess AT browning and metabolic homeostasis.

**Results:**

Propionate upregulated brown fat markers UCP1 and PGC1α in adipose tissue and mature adipocytes, particularly of mesenteric origin. Propionate exposure led to increased mitochondrial respiration and adenosine triphosphate production, primarily in mesenteric adipocytes, along with improved glucose uptake and reduced lipolysis and inflammation. In addition, propionate increased thermogenesis, glycolysis, and lipogenesis.

**Conclusion:**

The pronounced response of mesenteric AT to propionate underscores its potential as a therapeutic target for managing abdominal obesity and metabolic disorders.

Diet substantially affects metabolic health, with carbohydrates accounting for 45% to 65% of caloric intake, being vital for energy metabolism (1). Epidemiological evidence demonstrates that high fiber intake is associated with lower body weight (2). The composition of dietary carbohydrate quality affects adipocyte metabolism in humans and animals, suggesting an interplay between this diverse group of molecules and adipose tissue (AT) function (3). In the colon, carbohydrate that escapes digestion in the small intestine is fermented by the gut microbiota producing short-chain fatty acids (SCFAs): acetate, propionate, and butyrate (4). SCFAs activate free fatty acid receptors 2 and 3 (FFAR2; FFAR3) on a diverse number of tissues including ATs. Numerous studies have documented FFAR2 expression in human and mouse adipose tissues and cultured adipocytes (5-8). Conversely, FFAR3 detection in adipocytes varies, often showing minimal or undetectable levels (6). Activation of FFAR2 by SCFAs in AT modulates metabolic processes, including lipid metabolism, glucose homeostasis, adipocyte differentiation, and browning. Evidence suggests that FFAR2 engagement attenuates lipolysis by reducing free fatty acid (FFA) release (9). Further, FFAR2 may enhance glucose homeostasis through increased glucose uptake and activation of adenosine monophosphate–activated protein kinase (10). Evidence suggests that SCFAs reduce inflammation in AT through mechanisms involving not only adipocytes but also immune cells, including macrophages and T cells (11). SCFAs are also implicated in the induction of AT browning, evidenced by upregulation of thermogenic markers, elevated energy expenditure, and enhanced insulin sensitivity (12).

Among the SCFAs, propionate has the highest affinity for FFAR2 (13), with binding affinities ranging from high micromolar to low millimolar concentrations. Propionate is a preferred ligand for FFAR2, demonstrating a half maximal effective concentration of 250 to 500 μmol (14). Emerging evidence reveals that approximately 90% of propionate can be transported from the colon, passes the colonocytes and the viscera, and drains into the portal vein. Animal studies have demonstrated that an oral dose of propionate (333 μmol/kg) can increase portal vein concentrations to approximately 1300 μmol (15). The high concentration of propionate observed in the portal vein suggests that considerable metabolic effects could occur between the colon and liver (16). While limited studies have quantified portal SCFA concentrations, existing evidence has demonstrated that 3 mM propionate exposure for 24 hours in omental (17) and subcutaneous AT (18), as well as isolated adipocytes, enhanced FFAR2 expression and elicited anti-inflammatory effects without toxicity, supporting its safe application at higher concentrations. Furthermore, a 24-week human intervention trial involving 10 g/day of propionate supplementation confirmed the safety and revealed a significant reduction in abdominal adipose tissue, further supporting the therapeutic potential of propionate (19). This raises the possibility that increasing the concentration of gut-derived propionate may be a novel therapeutic approach for targeted modulation of abdominal AT to improve metabolic health (19).

Contemporary research highlights the critical role of AT distribution in determining metabolic risk, with abdominal AT being particularly significant (20). The fat depots are broadly classified into subcutaneous AT (SAT), which lies beneath the skin and muscle for energy storage, and visceral AT (VAT), located deep within the abdominal cavity near internal organs and substantially influencing metabolic dysfunctions such as insulin resistance (21). This effect is due to VAT's distinct metabolic activities (22) and inflammatory profiles (23). Within VAT, mesenteric adipose tissue (MAT) and omental adipose tissue (OAT) are differentiated by location and function. MAT surrounds the portal vein and other key structures and directly affects hepatic insulin sensitivity and systemic metabolic responses (24). In contrast, OAT, which interacts with the spleen and other abdominal organs, has a lesser effect on glucose metabolism. Research shows that removing MAT can greatly improve insulin sensitivity (25) underscoring its role as an independent determinant of metabolic health, whereas removing OAT has minimal effects (1).

AT also exhibits remarkable plasticity, particularly through the transdifferentiation of white adipocytes in white adipose tissue (WAT) into thermogenic brown/beige adipocytes, a process termed *“browning.”* (26). Beige adipocytes within WAT share properties with classic brown adipocytes including heat production and enhanced energy expenditure (27). Triggered by various stimuli including cold, hormones, and diet (28), the specific processes involved in browning, particularly the influence of dietary nutrients, remain underresearched, indicating an important area for future study.

Although studies increasing the intake of propionate reveal the effect on abdominal AT function (19), the underlying mechanisms and ability of propionate to modulate AT browning and metabolic homeostasis remain unclear. Therefore, this study aimed to detect whether propionate induces the browning of AT to elevate energy expenditure and regulate metabolic function, and to determine if these effects vary among ATs from different abdominal locations.

## Materials and Methods

### Experimental Participant Recruitment

Three AT samples from MAT, OAT, and SAT were collected from 40 patients who underwent open abdominal surgery for cholecystectomy or explorative laparotomy at Royal Surrey Hospital (ethical approval: CRO1396) and Hammersmith Hospital (ethical approval: 288071) in the United Kingdom for all experiments conducted in this study. The detailed data for the specific experiments carried out on the MAT, OAT, and SAT from each volunteer are listed in Table 1. All study participants received written and oral information before giving written informed consent for the use of the tissue.

**Table 1.** Summary of patient volunteer samples by experiment type

	Experiments	Patient volunteer No.
Adipose tissue		
	OCR and ECAR measurement	6
	Gene expression measurement	6-9
	Proinflammation response assay	9-10
Adipocyte		
	Lipid droplet size	5
	Gene expression measurement	6-9
	Glucose uptake assay	11-12
	FFA release	10
	Lipolysis experiments	8
	Proinflammation response assay	9-10

Each patient volunteer contributed mesenteric adipose tissue, omental adipose tissue, and subcutaneous adipose tissue samples for specific experimental analysis.

Abbreviations: ECAR, extracellular acidification rate; FFA, free fatty acid; OCR, oxygen consumption rate.

Ethical standards regarding the collection and use of human tissue and data were rigorously adhered to during this study, ensuring full confidentiality and compliance with ethical guidelines throughout the study.

### Adipose Tissue Culture, Adipocyte Isolation, and Culture

#### Adipose tissue culture

AT was immediately submerged in Dulbecco’s modified Eagle’s medium (DMEM)/F12 (Sigma-Aldrich D6421) and 1% Pen/Strep (Sigma-Aldrich P0781) solution and transferred to the laboratory at 4 °C. After the removal of fibrous material, AT was finely minced (20 mg) and cultured in DMEM/F12(Sigma-Aldrich D6421), 10% fetal bovine serum (Sigma-Aldrich F9665), and 1% Pen/Strep (Sigma-Aldrich P0781). ATs were cultured for 24 hours with either vehicle control (1 mM NaCl) or 1 mM propionate (Thermo Fisher A17440.0E) treatment, respectively, for the following experiments.

#### Adipocyte isolation

Finely minced AT was digested in collagenase buffer containing 2% bovine serum albumin (BSA) (Sigma-Aldrich A8806), 200 nM adenosine (PIA) (Sigma-Aldrich P4532), and 1 mg/mL type 1 collagenase (Thermo Fisher 31330-095) for approximately 40 minutes at 37 °C. Digested material was passed through a 250-μm strainer and centrifuged at 50*g* for 3 minutes to pellet the cells, creating 3 layers: free lipid, mature adipocytes, and digestion buffer. An 18-gauge needle and syringe were used to remove the free lipid and buffer.

#### Membrane setup for adipocyte culture

All membrane experiments were performed according to a previously published protocol (29) using 0.4-μm pore inserts. Transwells sized 6.5 mm (Costar-3413 and 3397) were used for all messenger RNA (mRNA) and imaging experiments; 24-mm Transwells (Costar-3412) were used for protein experiments. Transwells were placed upside down (the conical bottom facing up). Using a wide-bore tip, 30 μL/1 mL of packed human adipocytes (approximately 60 000/2 000 000) were pipetted onto 6.5 and 24 mm Transwells, respectively. The Transwells were lifted, inverted, and placed into a well containing 0.5 to 2 mL of culture medium (DMEM/F12, 10% fetal bovine serum, 1% Pen/Strep). The cells remained attached during the inversion. Cells were cultured for 24 hours with either vehicle control or 1 mM propionate treatment for the following experiments.

### Oxygen Consumption Rate and Extracellular Acidification Rate Measurements

Metabolic flux analyses were performed on ATs using a Seahorse XFe96 Extracellular Flux Analyzer (Seahorse Bioscience Inc) as described elsewhere (40). AT was placed and attached to Seahorse XFe96 FluxPak (PDL plates) (Agilent Technologies 103729-100). Media was changed to Seahorse XF DMEM assay medium (Agilent Technologies 103680-100) 1 hour before undertaking the assays. Basal, uncoupled, and nonmitochondrial cellular oxygen consumption rates (OCR), proton leak, and estimates of anaerobic glycolysis (extracellular acidification rates [ECAR]) were measured according to the manufacturer's protocol using DMEM assay medium containing 17.5 mM glucose and 1 mM pyruvate, pH 7.4. After equilibration, the XFe Cell Mito Stress Test was carried out using 54 nM oligomycin, 100 nM BAM15, and 22 nM rotenone/antimycin according to the Seahorse XF T Cell Metabolic Profiling Kit (Agilent Technologies 103772-100). Results were normalized to total protein content. Calculation of adenosine triphosphate (ATP) production rates using the Seahorse XF Analyzer was followed according to a previously published protocol (30).

### RNA Isolation, Reverse Transcription, and Quantitative Polymerase Chain Reaction

For AT, 100 mg of frozen tissue was homogenized in 1 mL TRIzol (Thermo Fisher 15596026). For adipocytes, after aspirating the medium, the cells were homogenized in 500 μL TRIzol. The TRIzol was pipetted up and down to dislodge all cells and incubated for 5 minutes followed by purification using Pure Link RNA Mini Kit (Thermo Fisher-12183018A). Isolated RNAs were reverse transcribed using the High-Capacity cDNA Synthesis kit (Thermo Fisher-4368814), and gene expression was measured using real-time polymerase chain reaction with TaqMan Universal Master Mix (Thermo Fisher-4304437) on a Quantstudio 7 Flex Real-Time PCR machine (Applied Biosystems). The relative expression levels of mRNA of FFAR2 (Hs00271142 s1), CEBPα (Hs00269972 s1), UCP1 (Hs00222453 m1), PPARγ (Hs01115513 m1), PGC1α (Hs00173304 m1), GLUT4 (Hs00168966 m1), tumor necrosis factor α (TNFα) (Hs00174128 m1), interleukin-6 (IL6) (Hs00174131 m1), FASN (Hs01005622 m1), PKM (Hs00761782 s1), and HK2 (Hs00606086 m1) were compared to the vehicle control and normalized to 18S (Hs03003631 g1), using the 2^−ΔΔCt^ formula.

### Adipocyte Lipid Measurement

Adipocytes were fixed for 15 minutes in 4% formaldehyde, stained with 25 μg/mL bodipy (ThermoFisher-D3922) and 2 μg/mL Hoechst 33 342 (ThermoFisher-H3570) for 30 minutes at room temperature (RT) and were subsequently washed with phosphate-buffered saline. Imaging was performed using a fluorescence microscope (Nikon-52634) at a magnification of 100×. The lipid area was measured using ImageJ software for quantitative analysis.

### Glucose Uptake Assay

Glucose uptake experiments were performed on membrane setup for adipocyte culture (MAAC)-cultured adipocytes. Adipocytes were cultured in culture medium (DMEM/F12, 2% BSA, 1% Pen/Strep) for 24 hours with either vehicle control or 1 mM propionate treatment. After serum-starving the cultured adipocytes for 6 hours in Krebs-Ringer Bicarbonate Buffer (KRBB) (25 mM HEPES (GIBCO)) supplemented with 2% fat-free BSA, the glucose uptake assay was performed using the Screen Quest Fluorimetric Glucose Uptake Assay Kit (36 500). Adipocytes were categorized into 4 treatment groups: The untreated group received no treatment; the insulin group was treated with 1 μM insulin; the control group received 1 μM insulin and vehicle control; and the propionate group was treated with 1 μM insulin and 1 mM propionate in the medium for 20 minutes. Cells were exposed to 2-deoxyglucose (2-DG) for 20 minutes at 37 °C. Following washing and cell lysis, 2-DG uptake was assessed by fluorescence at Ex/Em = 540/590 nm after incubating with the 2-DG Uptake Assay working solution for 30 minutes at RT.

### Free Fatty Acid Release

MAAC-cultured adipocytes were maintained in culture medium (DMEM/F12, 2% BSA, 1% Pen/Strep) for 24 hours with either vehicle control or 1 mM propionate treatment. FFA release detection was performed in the culture medium using the NEFA (nonesterified fatty foods) assay kit (FA115). A total of 10 μL of the appropriate standards and culture medium samples were transferred into separate wells; 200 μL buffer medium was then added to the wells, followed by a 10-minute incubation at RT. Subsequently, 400 μL of enzyme reagent was added to the wells and incubated for another 10 minutes at RT. The concentration was assessed by colorimetric absorbance at 700 and 550 nm, respectively.

### Lipolysis Experiment

The lipolysis experiment was performed on MAAC-cultured adipocytes. Adipocytes were cultured in culture medium (DMEM/F12, 2%BSA, 1%Pen/Strep) for 24 hours with either no treatment, vehicle control, or 1 mM propionate treatment. Lipolysis was stimulated by adding 100 nM isoproterenol (Sigma-I6504). Adipocytes were categorized into 4 treatment groups: The negative control group received no treatment; the positive control group was treated with 100 nm isoproterenol; the vehicle control group received 100 nm isoproterenol and 1 mM NaCl; and the propionate group was treated with 100 nm isoproterenol and 1 mM propionate in the medium. The cells were incubated at 37 °C, and 10 μL of the medium was collected at 90 minutes and 4 hours for glycerol analysis.

For the glycerol analysis, the procedure was followed according to the Sigma-Aldrich Glycerol Assay Kit (MAK1170). A total of 10 μL of the appropriate standards and culture medium samples were transferred into separate wells within a 96-well plate. The master reaction mix was prepared and mixed into the blank, standard, and sample wells followed by a 20-minute incubation at RT. The glycerol concentration was assessed by fluorescence at Ex/Em = 535/587 nm. Values were normalized to total protein.

### Proinflammation Response Assay

The proinflammation response assay was performed on ATs and adipocytes. After culture for 24 hours with different treatments, the media was collected and cytokine levels were measured according to the Human IL-6 DuoSet ELISA kit (Bio-Techne Ltd DY206, RRID:AB_2814717) and the Human TNF-alpha DuoSet ELISA kit (Bio-Techne Ltd DY210, RRID:AB_2848160). The procedure involved coating a 96-well plate with capture antibody (100 μL/well) and incubating overnight. After washing, reagent diluent (300 μL/well) was added for a 1-hour incubation, followed by a washing step. Culture media samples or standard (100 μL/well) were added, and the plate was incubated for 2 hours. After another washing step, detection antibody (100 μL/well) was added and incubated for 2 hours. Streptavidin–horseradish peroxidase (100 μL/well) was added and incubated for 20 minutes, followed by another wash step. Substrate solution (100 μL/well) was added and incubated for 20 minutes in the dark. The assay was concluded by adding a stop solution (50 μL/well). The concentration was assessed by colorimetric absorbance at 450 nm. Values were normalized to total protein.

### Total Protein

Lysis of the tissue and adipocytes was performed using radioimmunoprecipitation assay buffer followed by centrifugation and collection of the supernatant. The total protein evaluation was performed following the Pierce Modified Lowry Protein Assay Kit (Thermo Fisher 23240). A total of 40 μL of each standard and sample was pipetted into a 96-microplate well. Then, 200 μL of Modified Lowry Reagent was added to each well, and the microplate was incubated at RT for 10 minutes. A total of 20 μL of prepared 1X Folin-Ciocalteu Reagent was added to each well followed by a 30-minute incubation at RT. Subsequently, the plate was assessed by colorimetric absorbance at 750 nm.

### Statistical Analysis

Statistical analyses were performed using the SPSS 21.0 software and GraphPad Prism 7.04. Data were examined for normality according to the Shapiro-Wilk normality test. Analysis of gene expression levels and proinflammation response were assessed via paired 2-tailed t test (if parametric) and the Wilcoxon signed ranks test (if nonparametric). Analysis of the glucose uptake and lipolysis assay was performed via one-way analysis of variance (if parametric) or Kruskal-Wallis test (if nonparametric) followed by Tukey (if parametric) or Dunn (if nonparametric) multiple comparisons test to define statistically significant differences between individual groups. *P* values less than .05 were considered statistically significant.

## Results

### Patient Volunteer Characteristics

The patient volunteers were an average age of 67.33 ± 1.67 years, a weight of 75.41 ± 2.18 kg, and a height of 170.6 ± 1.55 cm. The average body mass index (BMI) of the participants was 25.96 ± 0.74. Detailed information on the patient volunteer is provided in Table 2.

**Table 2.** Characteristics of patient volunteers for adipose tissue collection

**Sex, n**	Female	15
	Male	25
**Age, y*^a^***		67.33 ± 1.67
**Height, cm*^a^***		170.6 ± 1.55
**Weight, kg*^a^***		75.41 ± 2.18
**BMI*^a^***		25.96 ± 0.74
**BP, mm Hg*^a^***	Systolic BP	134.2 ± 3.08
	Diastolic BP	76.25 ± 1.69
**HR, bpm*^a^***		76.6 ± 1.58
**Operations (n)**		
Whipple, pancreaticoduodenectomy		15
PPPD		13
LC		7
Laparoscopic deroofing of liver cysts		3
Liver wedge section		1
Mesh hernioplasty		1

Abbreviations: BMI, body mass index; BP, blood pressure; HR, heart rate; LC, laparoscopic **c**holecystectomy; PPPD, pylorus preserving pancreaticoduodenectomy.

^*a*^Mean and SEM.

### Propionate Increases the Browning Process and Enhances Thermogenic Capacity in Mesenteric Adipose Tissue

**Figure 1.**
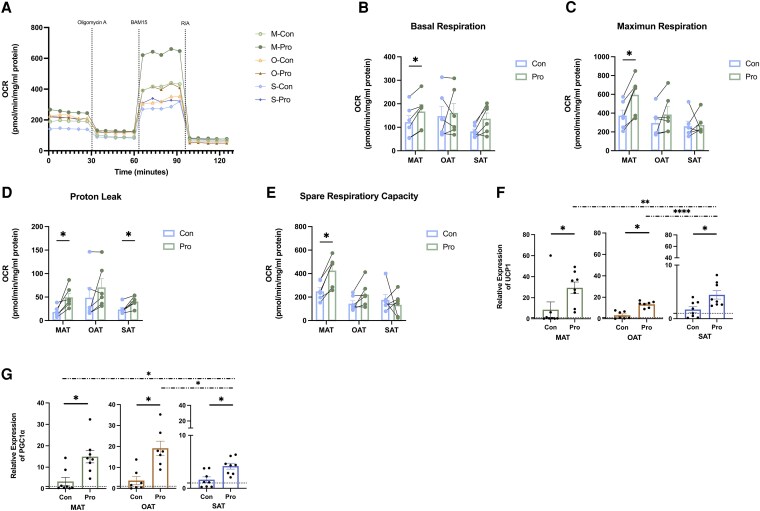
Effects of propionate on mitochondrial respiration in distinct adipose tissue depots. Seahorse bioanalysis and gene expression were performed on cultured AT treated for 24 hours with propionate (1 mM) or a vehicle control. A, A seahorse mito stress test was performed on AT to assess key parameters of mitochondrial respiration. Dotted lines indicate injections into media of the specific compounds: oligomycin A, BAM15, and rotenone/antimycin A (R&A). B, Basal respiration. C, Maximum respiration. D, Proton leak. E, Spare respiratory capacity (mean ± SEM, n = 6). F and G, Relative messenger RNA expression of UCP1 and PGC1α in AT in response to propionate (1 mM) treatment vs vehicle control (mean ± SEM, n = 5). ^#^*P* less than .05, propionate vs vehicle control; *P less than .05; ****P less than .0001. Abbreviations: AT, adipose tissue; M, mesenteric; O, omental; S, subcutaneous.

Propionate treatment elicited a significant augmentation in mitochondrial respiratory activity within human MAT (Fig. 1A), as evidenced by the significant elevation in basal respiration (Fig. 1B), maximal respiration (Fig. 1C), uncoupled OCR (proton leak) (Fig. 1D), and spare respiratory capacity (Fig. 1E) following 24-hour propionate exposure. While OAT displayed a similar trend of increased respiratory activity post treatment with propionate, no statistically significant differences were detected. In SAT, after 24-hour propionate exposure, a statistically significant elevation was observed solely in the uncoupled OCR (Fig. 1D).

Subsequent analysis provided further substantiation that propionate increases thermogenesis via analysis of expression of thermogenic genes within AT subtypes. Propionate treatment resulted in a marked upregulation of UCP1 (Fig. 1F) and PGC1α (Fig. 1G) both in MAT and SAT. MAT demonstrated a more pronounced elevation compared to cultures from the other AT depots.

### Propionate Increases the Glycolytic Flux in Adipose Tissue

**Figure 2.**
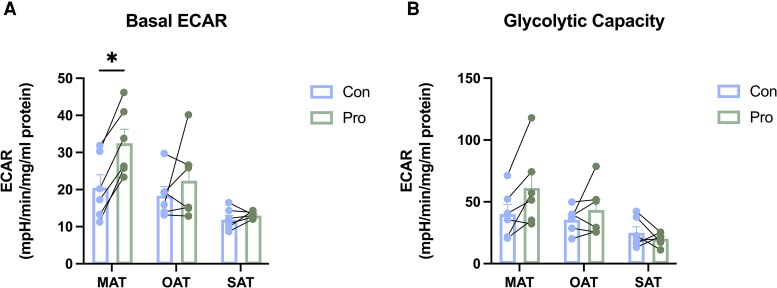
Effects of propionate on glycolytic flux in ATs. Seahorse bioanalysis was performed on cultured AT treated for 24 hours with propionate (1 mM) or a vehicle control. A, Basal extracellular acidification rate of AT. B, Glycolytic capacity (mean ± SEM, n = 6). *P less than .05. Abbreviations: AT, adipose tissue; Con, control group; M, mesenteric; O, omental; Pro, propionate group; S, subcutaneous.

Extracellular acidification rate (ECAR) is a measure of changes in extracellular pH that is considered to primarily reflect the media lactate concentration and is used as a cellular index of glycolysis. For MAT cultures, propionate significantly increased ECAR under basal conditions (Fig. 2A). MAT and OAT showed an increasing trend after oligomycin A, as well as mitochondrial uncoupler BAM15 injection (Fig. 2B), but no statistically significant differences were detected.

### Propionate Increases GlycoATP Generation in Mesenteric Adipose Tissue to Enhance Thermogenesis

**Figure 3.**
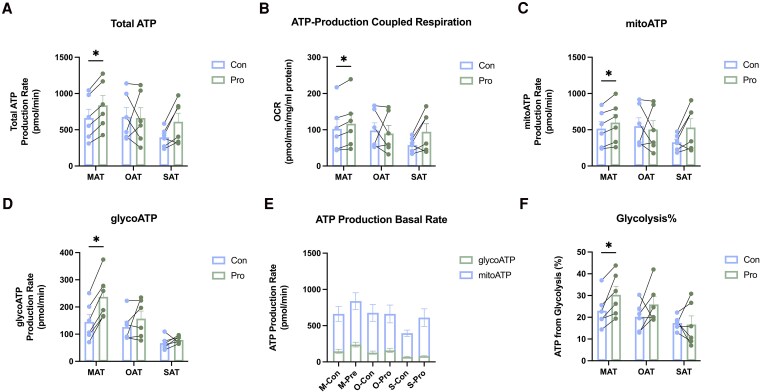
Effects of propionate on ATP generation. Seahorse bioanalysis was performed on cultured AT treated for 24 hours with propionate (1 mM) or a vehicle control. A, Total cellular ATP production rates. B, ATP-production coupled respiration. C, Mitochondrial cellular ATP production rates calculated the ATP generated from aerobic respiration. D, glycolytic ATP production rate calculated the ATP generated from anaerobic respiration. E, Cellular ATP production rates calculated for basal conditions. F, Percentage of glycolytic ATP production rate (mean ± SEM, n = 6). *P less than .05. Abbreviations: AT, adipose tissue; ATP, adenosine triphosphate; Con, control group; glycoATP, ATP generated from glycolytic pathway; M, mesenteric; mitoATP, ATP generated from mitochondrial pathway; O, omental; Pro, propionate group; S, subcutaneous.

Energy from glucose is provided predominantly via oxidative phosphorylation in the mitochondria (producing mitoATP) and glycolysis in the cytosol (producing glycoATP). The results showed that propionate treatment elicited a statistically significant increase in total ATP generation (Fig. 3A). Further, propionate significantly increased ATP-production coupled respiration (Fig. 3B), mitoATP (Fig. 3C), and glycoATP (Fig. 3D) in MAT. Quantitative analysis revealed that in MAT, ATP production attributable to glycolysis (glycoATP) was significantly enhanced by 7.47% after propionate treatment (Fig. 3F). OAT displayed a similar trend of increased glycolysis by 5.68% post treatment with propionate; no significant differences were detected (Fig. 3F). In SAT, no statistically significant differences were detected.

### Propionate Enhances Expression of Lipogenesis Genes and Decreases Proinflammation Response in Adipose Tissue

**Figure 4.**
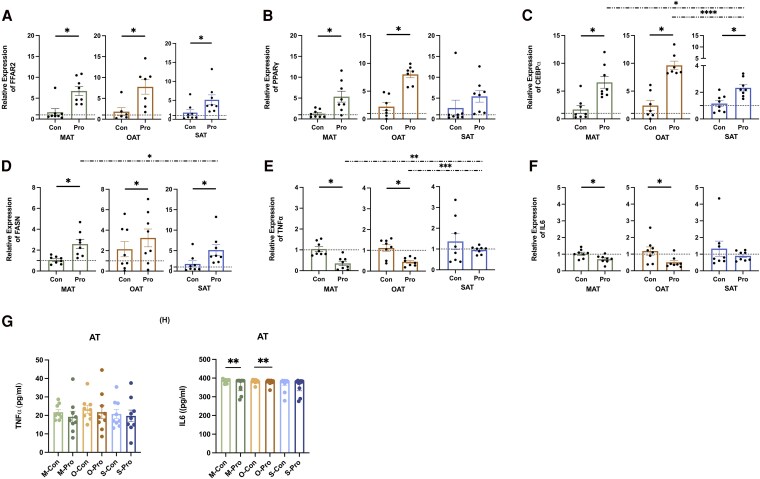
Effects of propionate on lipogenesis and proinflammation response in ATs. A to F, Relative messenger RNA expression of FFAR2, PPARγ, CEBPα, FASN, TNFα, and IL6 in AT in response to propionate (1 mM) treatment vs vehicle control (mean ± SEM, n = 6-9). G, TNFα release after 24 hours of treatment in AT (mean ± SEM, n = 10). H, IL6 release after 24 hours of treatment in AT (mean ± SEM, n = 9). ^#^*P* less than .05, ^##^*P* less than .01, propionate vs vehicle control; **P less than .01. Abbreviations: AT, adipose tissue; Con, control group; IL6, interleukin-6; M, mesenteric; O, omental; Pro, propionate group; S, subcutaneous; TNFα, tumor necrosis factor α.

The results showed that post propionate treatment, there was a statistically significant upregulation of FFAR2 in all 3 kinds of ATs (Fig. 4A). Propionate significantly increased PPARγ in MAT and SAT (Fig. 4B), CEBPα in OAT and SAT (Fig. 4C), and FASN in MAT and OAT (Fig. 4D). MAT demonstrated the highest increase in FASN compared to the other 2 ATs (Fig. 4D). Further, the results showed that propionate significantly decreased TNFα in MAT (Fig. 4E), and IL6 in MAT and OAT (Fig. 4F) but not in SAT. For the cytokine release, after 24 hours, propionate significantly inhibited the IL6 release in MAT and OAT (Fig. 4H), and no statistically significant differences were detected in TNFα (Fig. 4G).

### Propionate Increases Expression of Markers of the Browning Process, Glucose Uptake, and Glycolysis in Adipocytes

Since adipose tissue is composed of many cell types, to specifically investigate the cell-autonomous actions of propionate in adipocytes, adipocytes were successfully isolated from the 3 adipose tissue deposits. Measurements of the lipid droplet size across 3 types of adipocytes showed that mesenteric adipocytes had the smallest lipid area (MA: 33.63 ± 3.87 µm², untreated) compared to omental (OA: 37.95 ± 3.51 µm², untreated) and subcutaneous adipocytes (SA: 52.51 ± 6.96 µm², untreated), with a statistically significant difference observed between MA and SA. This pattern remained almost unchanged throughout the 24-hour culture period. Similar stability was observed following NaCl treatment (MA: 36.31 ± 1.58 µm², OA: 42.00 ± 2.23 µm², SA: 52.54 ± 5.03 µm²) and propionate treatment (MA: 34.32 ± 2.41 µm², OA: 41.13 ± 3.16 mm², SA: 51.40 ± 6.93 µm²). Detailed information is listed in Supplementary Fig. SA2 and Supplementary Table SA1 (31).

**Figure 5.**
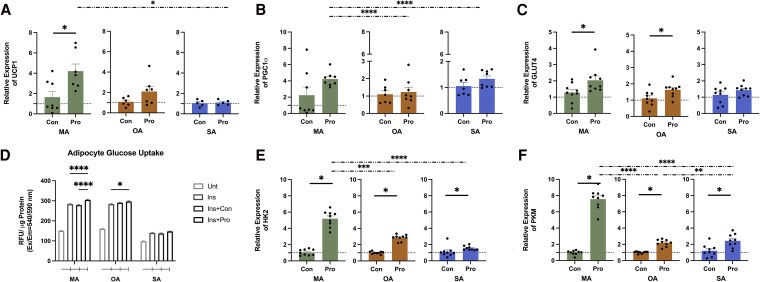
Effects of propionate on adipocyte browning, glucose uptake, and glycolysis. A to C, Relative messenger RNA (mRNA) expression of UCP1, PGC1α, and GLUT4 in adipocytes in response to propionate (1 mM) treatment vs vehicle control (mean ± SEM, n = 6-9). D, Glucose uptake in adipocytes. Ins + Pro: 1 μM insulin and 1 mM Propionate (mean ± SEM, n = 11-12). Unt: untreated; Ins: 1 μM insulin; Ins + Con:1 μM insulin and vehicle control. E and F, Relative mRNA expression of HK2 and PKM in adipocytes in response to propionate (1 mM) treatment vs vehicle control (mean ± SEM, n = 6-9). ^#^*P* less than .05, ^##^*P* less than .01, ^####^*P* less than .0001 propionate vs vehicle control; *P* less than .05 vs Unt group; *P less than .05, **P less than .01, ****P less than .0001. Abbreviations: A, adipocyte; M, mesenteric; O, omental; S, subcutaneous;.

Postpropionate treatment of isolated adipocytes from MAT induced a statistically significant upregulation of UCP1 mRNA expression (Fig. 5A). MA demonstrated the highest increase in UCP1 and PGC1α compared to the adipocytes prepared from OA and SA (see Fig. 5A and 5B).

Further, propionate treatment resulted in a significant upregulation of GLUT4 expression in MA (Fig. 5C). Consistent with the increased GLUT4 expression, insulin-stimulated glucose uptake was elevated with propionate treatment of adipocytes isolated from all the tested depots. Following propionate stimulation, the mature adipocytes consumed large amounts of glucose, as evidenced by the increased glucose concentration in the MA and OA (Fig. 5D). The results also showed that propionate significantly increased the expression of HK2 (Fig. 5E) and PKM (Fig. 5F) in MA and OA. MA demonstrated the highest increase in HK2 and PKM when compared to OA and SA.

### Propionate Increases Markers of Lipogenesis and Suppressed Lipolysis in Adipocytes

**Figure 6.**
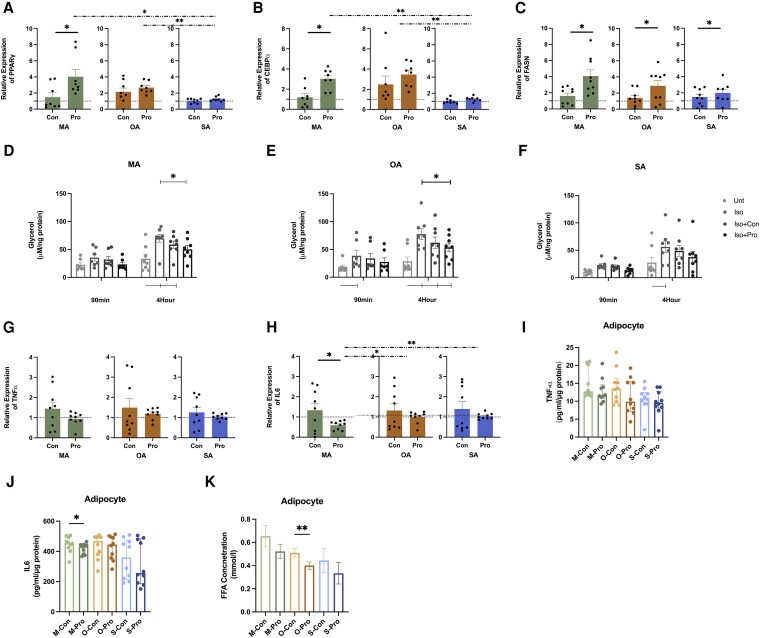
Effects of propionate on lipogenesis, lipolysis, and proinflammation response in adipocytes. A to C, Relative messenger RNA (mRNA) expression of PPARγ, CEBPα, and FASN adipocytes in response to propionate (1 mM) treatment vs vehicle control (mean ± SEM, n = 6-9). D to F, Isoproterenol-stimulated lipolysis in adipocytes cultured under membranes overnight. Iso + Con: 100 nm isoproterenol and vehicle control; Iso + Pro: 100 nm isoproterenol and 1 mM Propionate; Unt: untreated; Iso: 100 nm isoproterenol (mean ± SEM, n = 8). G and H, Relative mRNA expression of TNFα and IL6 in adipocytes in response to propionate (1 mM) treatment vs vehicle control (mean ± SEM, n = 6-9). I, TNFα release after 24 hours of treatment in adipocytes (median ± IQR, n = 9). J, IL6 release after 24 hours of treatment in adipocytes (median ± IQR, n = 10). K, Free fatty acid release after 24 hours of treatment in adipocytes (mean ± SEM, n = 10). ^#^*P* less than .05, ^##^*P* less than .01, propionate vs vehicle control; *P* less than .05 vsUnt group; *P less than .05, ***P less than .001, ****P less than .0001. Abbreviations: A, adipocyte; IL6, interleukin-6; IQR, interquartile range; M, mesenteric; O, omental; S, subcutaneous; TNFα, tumor necrosis factor α.

Postpropionate treatment instigated a statistically significant upregulation of CEBPα in MA (Fig. 6A) and FASN in all adipocytes from all the depots tested (Fig. 6C). MA showed the highest propionate-stimulated increase in CEBPα, PPARγ, and FASN (see Fig. 6A-6C). Furthermore, after 4 hours of incubation, propionate-treated MA- and OA-conditioned media showed significant decreases in glycerol concentration after isoproterenol stimulation (Fig. 6D and 6E) but not in SA (Fig. 6F). The reduced levels of glycerol released indicate that propionate treatment inhibits isoproterenol-stimulated lipolysis in MA and OA (Fig. 6K). For the proinflammation response, propionate treatment significantly decreased the IL6 expression level (Fig. 6J) release in MA.

## Discussion

Propionate, a gut-derived SCFA, acts as a dual-action metabolic regulator by enhancing abdominal AT function and metabolic homeostasis (19). AT distribution in the abdominal region varies, with each type playing distinct roles in maintaining this homeostasis. Brown AT (BAT) activation promotes thermogenesis and energy expenditure, and maintains metabolic function. White adipocytes within WAT transdifferentiate into brown-like adipocytes termed *beige* or *brown-in-white* (BRITE) under stimuli such as prolonged cold exposure, β-adrenergic agonist treatment, or additional nutrients, through a process termed *“browning.”* It has been recently shown that browning is a specialized process that results in elevated expression of the thermogenic protein UCP1 and increased heat dissipation and energy expenditure (32). Such findings underscore the importance of adipocyte browning in the modulation of metabolic functions. While previous studies have demonstrated the influence of propionate on abdominal AT, its role in adipocyte browning and varying effects on different adipose types have yet to be fully clarified. This study offers new insights by showing that propionate not only promoted metabolic function but also enhanced the browning of adipocytes from WAT and increased thermogenic pathways, thereby enhancing energy expenditure, with a marked effect on MAT.

Propionate enhanced the browning process in AT resulting in upregulated expression of UCP1 and the metabolic coactivator PGC1α (33), especially in MAT. Thermogenesis, facilitated by UCP1, is characterized by increased energy and oxygen consumption, as indicated by elevated OCR, a direct measure of mitochondrial respiration. Functionally, UCP1 facilitates a proton leak across the inner mitochondrial membrane, uncoupling substrate oxidation from ATP synthesis, which in turn generates heat (34). After propionate treatment, an upsurge in proton leaking signifies an elevation in thermogenic activity, which results in augmented energy expenditure (35). Meanwhile, the elevated basal respiration indicates that propionate amplifies the baseline metabolic rate. Evaluation of maximal respiration revealed that propionate could stimulate mitochondrial respiratory capacity, serving dual purposes of energy and heat generation. Lastly, an increase in spare respiratory capacity suggests that propionate supplementation improves AT capacity to rapidly increase respiration, thus meeting increased energy demands alongside enhanced heat production. In addition, propionate also enhanced PPARγ and CEBPα gene expression to facilitate the browning process. PPARγ stimulation stabilizes the expression of PRDM16 mRNA, and the induced PGC1α interacts with PRDM16 to promote BAT-specific gene expression (36). CEBPα regulates adipogenesis alongside PPARγ to stimulate UCP1 expression (37). No statistically significant differences in UCP1 and PGC1α expression were observed among the 3 types of ATs under basal conditions; however, propionate treatment significantly upregulated their expression in all 3 ATs, with the most pronounced increase observed in MAT compared to the other two. Therefore, our findings show that propionate significantly promotes AT browning, particularly in MAT, as evidenced by elevated levels of browning marker expression and thermogenic activity.

Propionate boosts energy production by enhancing glycolysis in AT. Browning-dependent energetic processes necessitate a readily available fuel supply, which includes glucose and FAs. ECAR reflects extracellular pH and serves as a cellular index of glycolysis. An increase in basal ECAR on propionate addition suggests enhanced glycolysis, potentially fueling thermogenesis. Additionally, increasing glycolytic capacity suggests propionate amplifies glycolysis under metabolic demand or stress. Furthermore, the elevated expression of FASN to boost FA synthesis reinforces the role of glucose as a substrate for FA production, with propionate facilitating this process and additionally fueling thermogenesis (38).

Evidence from multiple studies shows that beige adipocytes, even though they express UCP1, also dissipate energy through processes that consume ATP (39, 40). Glucose, as the energy source, plays a crucial role in ATP synthesis through its breakdown. Glucose can first be metabolized into pyruvate, which can then enter the oxidative phosphorylation pathway within the tricarboxylic acid cycle in mitochondria to generate ATP (mitoATP) (41), or be directly converted to acetate to generate ATP (glycoATP) in the cytoplasm. Our findings showed that propionate could upregulate both mitoATP and glycoATP pathways. This modulation of glucose metabolism by propionate enhances ATP synthesis and, notably, elevates the proportion of glycoATP, corroborating the role of propionate in promoting browning through glucose utilization.

Propionate mediates anti-inflammatory effects that may play a role in supporting browning activity. TNFα impairs UCP1 gene expression in brown adipocytes in vitro. Compared with WAT, BAT from mice fed a high-fat diet tends to show markedly lower immune cell-enriched mRNA expression and macrophage infiltration, suggesting that BAT resists obesity-induced inflammation (42, 43).

In terms of volumetric occupancy, adipocytes are predominant in AT, comprising more than 90% of the tissue volume. This predominance is due to their unique morphology, characterized by large lipid droplets that occupy up to 90% of the cell volume, with the remaining cell structure including a thin cytoplasmic layer, peripherally situated nucleus, and relatively underdeveloped mitochondria (44). Recent studies have also shown that although immune cells are involved in some aspect of the tissue remodeling that occurs during WAT browning in humans after cold treatment, there are likely no significant relationships between the change in macrophage numbers and the change in UCP1 protein abundance (45). Therefore, further insight focused on the adipocyte is necessary.

Our study corroborates that the administration of propionate enhances UCP1 gene expression in isolated mesenteric adipocytes, consistent with the trends observed in our tissue-level results. This finding is supported by other studies that SCFA could increase thermogenic capacity in adipocytes (46, 47). Although statistical significance was not reached, mesenteric adipocytes exhibited the highest expression levels of UCP1 and PGC1α among the 3 AT depots examined. These morphological and functional differences suggest that MAT may have a greater capacity for browning compared to other depots. This observation is further supported by the fact that MAT displayed the smallest lipid area, suggesting a unique metabolic profile. Previous studies have shown that brown adipocytes are typically characterized by a smaller diameter compared to white adipocytes (48). Taken together, our results indicate that MAs represent a distinct cell type, and propionate appears to activate key factors in regulating energy homeostasis, further enhancing the browning process. As supported by previous research (49), this process is primarily mediated via FFAR2. These effects of propionate are most pronounced both in MAT and MA, indicating a site-specific response to metabolic modulation, such as obesity and diabetes.

The results obtained with isolated adipocytes further demonstrate that propionate enhances thermogenesis by increasing glucose uptake and glycolysis, thereby promoting adipocyte browning. Our data highlight the necessity of glucose assimilation and subsequent glycolytic activity for thermogenic processes in adipocytes. The study observed an increased GLUT4 expression and a significant increase in glucose uptake by mature adipocytes on propionate exposure. This suggests that propionate could activate glucose uptake that will contribute to fueling thermogenesis. Additionally, our results showed an increase in the expression of key glycolytic enzymes, HK2 and PKM, markers of enhanced glycolytic activity, which are essential for the initial and final steps of the glycolysis pathway (50). Such metabolic actions culminate in the promotion of overall energy expenditure. Importantly, these propionate-mediated effects are particularly evident in MAT, indicating a targeted response to this metabolic intervention. Although prior research has suggested that glucose could account for only 2% to 16% of the energy source for thermogenesis (51), the contribution through oxidation of FAs synthesized (52) de novo from glucose and other lipogenic substrates (eg, lactate) (53) could be considerably larger. The further results from adipocytes showed a similar trend in increased PPARγ, CEBPα, and FASN, indicating that propionate is a key modulator of lipid synthesis in distinct adipose depots. Propionate treatment decreased FFA release from adipocytes, suggesting a reduction in lipolysis and enhanced FFA utilization. Despite the absence of insulin, which normally increases lipolysis and FFA levels (54), propionate treatment still lowers FFA release. This indicates that propionate via promoting FA utilization may also contribute to stimulating the browning of adipocytes.

In conclusion, propionate serves as a significant metabolic regulator, stimulating energy expenditure by modulating glucose metabolism to enhance both thermogenic activity and ATP production within the AT, especially for MAT. Our results show that by regulating the expression of PPARγ, CEBPα, and FASN, propionate promotes healthy lipid storage and de novo lipogenesis, decreases inflammation, and enhances lipid-buffering capacity, which together improves systemic insulin sensitivity. This supports earlier studies that demonstrate propionate can help to reduce insulin resistance locally and systematically. These effects are further supported by propionate actions to maintain the lipid size in different kinds of adipocytes.

Second, propionate treatment attenuates lipolytic activity within the adipocyte. This may be due to FFAR2's ability to inhibit hormone-sensitive lipase, a mediator of lipolysis (55). Abundant FFA production from the dysregulation of lipolysis within adipocytes could mediate a positive feedback loop leading to more lipolytic stimulation within the cell (56). Overproducing FA influx into the liver and muscles leads to the formation of diacylglycerols and ceramides, which cause negative effects including reduced insulin release and promoting disease progression (56). However, FFAR2 activation would eliminate the overproduction of FFA from excessive lipolysis, thus, mitigating insulin resistance.

Third, propionate can inhibit inflammation. TNFα and IL6 can both directly inhibit FFA uptake and lipogenesis and stimulate of FFA release via lipolysis (57). Further, TNFα and IL6 exert long-term inhibitory effects on the expression of many proteins that are required for insulin-stimulated glucose uptake in adipocytes, such as insulin receptor substrate-1 (IRS-1) and GLUT4 (58). This results in insulin resistance not only in the adipocytes and immune cells included in the AT, but also distant organs and tissues.

The distinct response of MAT to propionate across 3 kinds of AT may be for the following reasons: MAT exhibits characteristics distinct from typical visceral fats, including a reduced macrophage presence, indicating less inflammatory activity (59). Second, MAT is characterized by higher adipogenic potential and contains lower quantities of fat-associated lymphoid clusters and β cells (60), highlighting its unique role in AT function and potential implications in metabolic regulation (25, 61). Third, MAT is connected to the large intestine, and the removal of MAT connected to the large intestine could also change the microbiota of small and large intestine contents (62). Excision of MAT may alter the intestinal microbiota, affecting both small and large intestinal environments. This investigation reveals that propionate's actions in MAT are greater than other depots, implying a divergent functional role for MAT compared to conventional visceral fat. OAT showed a similar response to propionate compared to MAT, whereas SAT did not. This is potentially because OAT, as another type of VAT, exhibited similar characteristics, such as a higher FFA uptake rate and a higher triglyceride turnover rate compared to SAT. Furthermore, the metabolic differences in OAT as a form of VAT may explain why propionate affects OAT but not SAT.

This study’s limitations include a slightly above-normal BMI in patient volunteer and a small sample size (7-12 participants), which may limit generalizability. It notes increased mitochondrial activities during adipose browning, though the specific mechanisms remain unclear. While this study examined propionate's effects across adipose depots, sex-specific responses were not evaluated. Given the current lack of evidence regarding sex differences in propionate's mechanisms of action in AT, future investigation is warranted. Despite these, the research is pivotal in advancing propionate's potential to enhance AT energy expenditure, meriting further investigation.

## Conclusion

Overall, we found that propionate enhances thermogenesis through glycolysis, boosts energy metabolism genes and glucose uptake, and reduces insulin resistance via lipolysis and inflammation. This could substantially benefit MAT function, potentially decreasing disease risk associated with MAT. Our findings provide a key contribution to understanding the physiological processes relevant to metabolic health affected by propionate. As such, exploration of the health benefits of propionate on AT provides an important public health solution to abdominal obesity, which underlies the development of metabolic diseases.

## Data Availability

Data described in the manuscript, code book, and analytic code will be made available on request.

## Abbreviations

2-DG: 2-deoxyglucose
AT: adipose tissue
ATP: adenosine triphosphate
BAT: brown adipose tissue
BMI: body mass index
BP: blood pressure
BSA: bovine serum albumin
ECAR: extracellular acidification rate
FFAs: free fatty acids
FFAR: free fatty acid receptor
HR: heart rate
IL6: interleukin-6
LC: laparoscopic cholecystectomy
MA: mesenteric adipocyte
MAAC: membrane setup for adipocyte culture
MAT: mesenteric adipose tissue
mRNA: messenger RNA
OA: omental adipocyte
OAT: omental adipose tissue
OCR: oxygen consumption rate
PPPD: pylorus preserving pancreaticoduodenectomy
RT: room temperature
SA: subcutaneous adipocyte
SAT: subcutaneous adipose tissue
SCFAs: short-chain fatty acids
TNFα: tumor necrosis factor α
VAT: visceral adipose tissue
WAT: white adipose tissue
